# Clinical Outcomes and Quality of Life Following Different Mesh and Fixation Methods in Inguinal Hernia Repair: A Retrospective Registry-Based Study

**DOI:** 10.7759/cureus.96095

**Published:** 2025-11-04

**Authors:** Suphakarn Techapongsatorn

**Affiliations:** 1 Surgery, Navamindradhiraj University, Bangkok, THA

**Keywords:** anatomical mesh, flat mesh, patient-reported outcome measures, primary inguinal hernia, quality of life, totally extraperitoneal repair

## Abstract

Background: The choice of mesh in totally extraperitoneal (TEP) inguinal hernia repair remains a subject of debate, particularly regarding the optimal mesh shape. While anatomical and flat meshes are widely used, few studies have rigorously compared the impact of mesh shape while standardizing other surgical factors like fixation. Consequently, there is limited evidence to quantify the clinical and economic value of potential early recovery benefits. We aimed to determine if anatomical mesh shape provides superior early patient-reported outcomes compared to flat mesh when a standardized metallic fixation technique is used in both groups.

Methods: This retrospective cohort study included 80 patients undergoing elective TEP inguinal hernia repair. Patients were categorized to receive either a three-dimensional anatomical mesh (n=39) or a standard flat mesh (n=41), with both groups undergoing standardized metallic tack fixation. The primary outcomes were patient-reported outcome measures (PROMs) assessed at one and six months postoperatively. Instruments included the validated Thai version of the Carolinas Comfort Scale (TCCS) for hernia-specific symptoms and the EQ-5D-5L with a Thai value set for health-related quality of life (HRQoL).

Results: At the one-month follow-up, the anatomical mesh group demonstrated significantly better outcomes, with lower mean scores across all TCCS domains - Pain (7.77 ± 0.97 vs. 10.14 ± 0.96), Mesh Sensation (7.02 ± 0.92 vs. 9.17 ± 1.14), and Movement (6.95 ± 0.89 vs. 8.91 ± 1.08) - and a higher EQ-5D-5L utility score (0.819 ± 0.019 vs. 0.784 ± 0.016; p < 0.001 for all). By six months, there were no statistically significant differences between the groups in either TCCS domains or EQ-5D-5L scores.

Conclusions: When the fixation method is standardized, the anatomical shape of the mesh is associated with a significantly better quality of life and fewer symptoms during the first month of recovery, a benefit that is transient. This study quantifies the value of early postoperative recovery, providing crucial evidence for a trade-off analysis between the higher cost of anatomical mesh and its tangible, short-term clinical benefits. These findings are essential for facilitating informed, value-based decisions in surgical practice.

## Introduction

Inguinal hernia is one of the most common surgical conditions, with a lifetime risk of 27% in men, and surgery remains the only definitive treatment [1]. Over the decades, surgical techniques have evolved from tension-based suture repairs to tension-free mesh repairs, such as the Lichtenstein technique, which significantly reduced recurrence rates [2]. The latest advancement is minimally invasive surgery, particularly the totally extraperitoneal (TEP) technique. TEP offers several advantages, including reduced postoperative pain, faster recovery, and, crucially, avoidance of entry into the peritoneal cavity, which lowers the risk of visceral injury and adhesions compared to the transabdominal preperitoneal (TAPP) approach [3].

The cornerstone of a successful TEP repair lies in strict adherence to Stoppa’s principle, which emphasizes reinforcing the entire myopectineal orifice (MPO), the anatomical weak point common to all types of groin hernias, including direct, indirect, and femoral. Understanding the dimensions of the MPO is critical for selecting appropriate mesh size to ensure full coverage and prevent recurrence. Anatomical studies, including those conducted in the Thai population, have confirmed that a standard 10 × 15 cm mesh provides sufficient coverage of the MPO, with average dimensions around 7 cm in width and 6 cm in height. This anatomical foundation supports mesh choice in the current investigation and is consistent with other studies that highlight the importance of tailored mesh design and coverage to improve outcomes in minimally invasive groin hernia repair [4-9].

However, an ongoing debate in the surgical community is the selection of the optimal mesh type. Beyond the comparison of heavyweight versus lightweight meshes, attention has now turned to the shape of the mesh. This involves comparing the traditional flat mesh, which is flexible but may sometimes require fixation, with the anatomical mesh, which is designed with a three-dimensional shape to conform to the anatomy of the MPO and is often intended to reduce the need for fixation [10,11].

In the modern era, the measure of surgical success has shifted. Traditional metrics like recurrence rates are no longer sufficient. The focus has moved towards patient-centered outcomes, such as chronic pain, quality of life, and satisfaction. Consequently, patient-reported outcome measures (PROMs) have become essential tools for genuinely assessing the patient's experience [12]. These can be divided into generic instruments, like the EQ-5D-5L, and disease-specific instruments, like the Carolinas Comfort Scale (CCS) [13].

Despite the use of various mesh types, to date, more than 10 comparative studies have investigated different mesh configurations in laparoscopic inguinal hernia repair, but only a few have directly compared anatomical and flat meshes. Therefore, this study aims to compare patient-reported outcomes at one and six months after TEP inguinal hernia repair using anatomical mesh versus standard flat mesh.

## Materials and methods

Study design and population 

Data were collected retrospectively from January 2022 to December 2024 at Vajira Hospital. Eligible patients were adults (age > 18 years) diagnosed with a primary, reducible inguinal hernia scheduled for elective TEP repair, who were fit for general anesthesia. Exclusion criteria included recurrent hernias, incarcerated hernias, previous lower abdominal surgery, or contraindications to the TEP procedure. The study was approved by the Institutional Review Board for Human Research, and all patients provided written informed consent before participation.

Surgical technique and study groups 

All patients underwent a standardized TEP procedure under general anesthesia. Patients were placed in the Trendelenburg position. A sub-umbilical incision was made for the camera port, with two additional small incisions in the midline above the pubis for instrument ports. The preperitoneal space was created using a balloon dissector, and dissection was performed to identify the entire MPO, including key anatomical structures like Cooper's ligament and the inferior epigastric vessels, before reducing the hernia sac [6].

As this was a retrospective study, patients were not randomised but were retrospectively categorized according to the mesh used. The choice of mesh depended on surgeon preference, mesh availability, and patient anatomical suitability. Group A received a three-dimensional anatomical mesh, either Bard™ 3DMax™ Mesh (BD, USA) or Parietex™ Anatomical Mesh (Medtronic, USA), while Group B received a flat macroporous polypropylene mesh (Parietene™ Mesh, Medtronic, USA). Both the anatomical and flat meshes measured 10 × 15 cm, providing adequate coverage of the MPO and corresponding to the standard size used for TEP repair. All meshes in both groups were fixated using metallic tackers to ensure proper positioning and prevent migration.

Outcome measures and data collection 

The primary outcomes were scores from PROMs collected at one and six months (medium-term) post surgery. The instruments used were the CCS, a hernia-specific questionnaire consisting of 23 items that assess symptoms in three main domains - pain, mesh sensation, and movement - across eight different activities. Scoring is performed on a Likert scale from 0 (no symptoms) to 5 (disabling symptoms), with lower scores indicating better outcomes [13]. This study used the Thai version of CCS (TCCS), which has been validated and shown to have excellent reliability (Cronbach’s alpha = 0.89) for measuring quality of life in Thai patients undergoing hernia repair [14].

The EQ-5D-5L is a generic health-related quality of life (HRQoL) questionnaire measuring five dimensions: mobility, self-care, usual activities, pain/discomfort, and anxiety/depression, each with five severity levels. The results from these five dimensions are converted into a utility index score using the Thai value set. Scores range from less than 0 (a state worse than death) to 1 (perfect health), with higher scores indicating better quality of life [15].

Statistical analysis 

All statistical analyses were performed using Stata version 16.1 (StataCorp LLC, USA). Descriptive statistics were used to describe baseline patient characteristics. Chi-squared or Fisher's exact test was used for categorical data, and independent samples t-test or Mann-Whitney U test was used for continuous data to compare baseline characteristics between the two groups. PROMs scores were compared between groups at each time point using the independent samples t-test or Mann-Whitney U test as appropriate. A p-value of < 0.05 was considered statistically significant.

## Results

A total of 80 patients were enrolled in the study, with 39 in the anatomical mesh group and 41 in the flat mesh group. As shown in Table 1, the baseline characteristics of the two groups were similar, with no statistically significant differences in age, BMI, or the prevalence of comorbidities such as hypertension, diabetes, or heart disease (p > 0.05 for all variables). Furthermore, the rates of early postoperative complications, such as hematoma or seroma, and length of hospital stay were not different between the two groups (Table 2).

**Table 1:** Baseline demographic and clinical characteristics of patients COPD: Chronic obstructive pulmonary disease

Variable	Anatomical Mesh Group (n = 39)	Flat Mesh Group (n = 41)	p-value
Age (years)	62.5 (13.1)	61.8 (14.5)	0.812
BMI (kg/m^2^)	22.69 (2.02)	22.95 (1.91)	0.557
Sex (Male)	35 (89.7%)	39 (95.1%)	0.361
Underlying disease			
- Hypertension	20 (51.3%)	23 (56.1%)	0.666
- Diabetes mellitus	12 (30.8%)	6 (14.6%)	0.118
- Heart disease	6 (15.4%)	8 (19.5%)	0.627
- COPD	2 (5.1%)	3 (7.3%)	0.672
- Prostatic disease	4 (10.3%)	5 (12.2%)	0.812

**Table 2:** Postoperative outcomes and complications

Variable	Anatomical Mesh Group (n = 39)	Flat Mesh Group (n = 41)	p-value
Length of stay (days)	4.67 (8.44)	3.71 (2.63)	0.49
Hematoma	2 (5.1%)	0 (0.0%)	0.142
Seroma	4 (10.3%)	5 (12.2%)	0.784
Urine retention	1 (2.6%)	1 (2.4%)	0.971

The results of the PROMs assessment at one and six months post surgery are presented in Table 3. At the one-month follow-up, there were statistically significant differences between the two groups. The anatomical mesh group had lower scores on the CCS, indicating fewer symptoms, across all domains: Pain, Mesh Sensation, and Movement (p < 0.001 for all). Similarly, the anatomical mesh group reported significantly better overall quality of life, with a mean EQ-5D-5L utility score of 0.819 compared to 0.784 for the flat mesh group (p < 0.001).

**Table 3:** Patient-reported outcomes at one and six months post surgery CCS: Carolinas Comfort Scale

Outcome	Anatomical Mesh Group (n = 39)	Flat Mesh Group (n = 41)	p-value
At one month			
- CCS - Pain	7.77 (0.97)	10.14 (0.96)	<0.001
- CCS - Mesh Sensation	7.02 (0.92)	9.17 (1.14)	<0.001
- CCS - Movement	6.95 (0.89)	8.91 (1.08)	<0.001
- EQ-5D-5L Utility	0.819 (0.019)	0.784 (0.016)	<0.001
At six months			
- CCS - Pain	2.35 (0.80)	2.48 (0.85)	0.276
- CCS - Mesh Sensation	2.22 (0.92)	2.39 (0.94)	0.348
- CCS - Movement	2.28 (0.88)	2.41 (0.90)	0.391
- EQ-5D-5L Utility	0.850 (0.021)	0.853 (0.022)	0.66

At the medium-term follow-up, the significant differences observed at one month had disappeared. There was no statistically significant difference between the anatomical and flat mesh groups in CCS scores for Pain (p = 0.236), Mesh Sensation (p=0.581), or Movement (p = 0.733). Similarly, the EQ-5D-5L utility scores for both groups were nearly identical (0.850 vs. 0.853, p = 0.660), indicating that the overall quality of life of patients in both groups was comparable in the medium term.

## Discussion

This study compared patient-reported outcomes between anatomical and flat mesh in TEP inguinal hernia repair. The key findings are: (1) at one month post surgery, anatomical mesh was associated with better local hernia-specific symptoms (lower CCS scores) and overall quality of life (higher EQ-5D-5L score), and (2) at medium term, these differences resolved, and outcomes for both groups were comparable.

The seemingly clear benefit in the first month is an important finding of this study. Both the disease-specific instrument (CCS) and the generic instrument (EQ-5D-5L) showed better results for anatomical mesh, suggesting greater patient comfort and quality of life during early recovery. One hypothesis to explain this benefit is that the preshaped three-dimensional design of the anatomical mesh conforms better to the MPO, reducing folding, tension, and irritation [16], which leads to lower CCS scores (fewer symptoms) for Pain, Mesh Sensation, and Movement [16]. This improved integration may also contribute to higher overall quality of life as measured by EQ-5D-5L.

Meanwhile, the higher EQ-5D-5L score in the anatomical mesh group complements the better CCS results, suggesting a consistent benefit across both disease-specific and generic measures. This may reflect not only reduced local discomfort but also greater confidence in resuming daily activities, as the anatomical mesh’s design minimizes folding and tension. The “Usual Activities” and “Anxiety” dimensions of EQ-5D-5L likely capture these broader effects. This finding underscores the value of using multiple PROM instruments to gain a comprehensive understanding of patient experience.

The convergence of outcomes for both groups at medium-term points to the body’s biological process of adapting to a foreign object. Over time, the body forms fibrosis and new tissue that encapsulates and integrates with the mesh, making both types of mesh a part of the strengthened abdominal wall. This process tends to diminish the initial differences between the two mesh types, as tissue remodeling and fibrosis create stable integration for both. This result is consistent with other studies comparing lightweight and heavyweight meshes, which often find that despite early differences, long-term quality of life and pain are usually similar [17,18]. The study extends this concept to differences in mesh shape.

This study's finding that anatomical mesh resulted in better local symptoms in the first month is consistent with studies suggesting that preshaped meshes can reduce postoperative pain and discomfort. This benefit may be due to improved anatomical conformity that minimizes folding, tension, and irritation. Additionally, differences in fixation methods may influence early pain; in this study, all meshes, including flat meshes, were fixated using metallic tacks to Cooper’s ligament to ensure stability and prevent migration, which can be a source of discomfort despite careful placement.

Similar considerations have been highlighted in studies comparing anatomical and flat meshes in TEP repair. Research indicates that anatomical meshes without fixation can achieve comparable outcomes to flat meshes with fixation, despite higher upfront costs [19]. Additionally, studies have reported benefits such as shorter operative time and fewer complications [20]. These findings support the potential clinical advantages of anatomical mesh and its value in cost-effectiveness analyses. This aligns with evidence that anatomical designs can reduce or eliminate the need for fixation, helping to minimize related postoperative discomfort [19].

The most critical issue arising from these results is the consideration of clinical and health economic cost-effectiveness. Anatomical meshes are generally more expensive than standard flat meshes. The question then becomes whether the added investment is justified by the benefit of improved early postoperative symptoms and overall quality of life, especially when the 6-month outcomes are identical. The answer to this question is crucial for surgeons and hospital administrators in making decisions about resource allocation and determining the most cost-effective treatment guidelines.

The main strengths of this study are the use of two validated PROM instruments to measure different dimensions of outcomes, and particularly the use of the TCCS, which has been specifically validated for the Thai population, making the data highly reliable in the context of Thailand.

However, this study has some limitations. First, it was conducted at a single institution, which may limit the generalizability of the results to other populations. Second, the number of patients is relatively small, and the six-month follow-up period may not be long enough to assess very long-term outcomes, such as recurrence rates or chronic pain that might develop one-two years post surgery. Finally, the different mesh fixation methods between the two groups could be a confounding factor affecting the results.

To confirm these findings, a multicenter randomized controlled trial (RCT) with a larger sample size and a longer follow-up period (e.g., one-two years) is needed to accurately assess recurrence rates and chronic pain. Future research should also standardize the mesh fixation method across all groups or randomize the fixation method to clearly separate the effects of mesh shape from the effects of the fixation method. Moreover, such large-scale studies should evaluate not only clinical outcomes but also the cost-effectiveness of anatomical mesh to provide robust evidence for guiding surgical practice and informing policy decisions.

## Conclusions

For patients undergoing TEP inguinal hernia repair, the choice between anatomical and flat mesh reflects a trade-off in the early recovery phase. Anatomical mesh may offer better overall quality of life and fewer local symptoms at one month. These differences are transient, as the outcomes of both groups become comparable by six months. Anatomical mesh may be preferred when early postoperative recovery and patient comfort are clinical priorities, whereas flat mesh remains a cost-effective alternative with comparable outcomes at medium term, considering the balance between the benefits and costs of each type of prosthetic material.
